# Single cell and bulk RNA expression analyses identify enhanced hexosamine biosynthetic pathway and O-GlcNAcylation in acute myeloid leukemia blasts and stem cells

**DOI:** 10.3389/fimmu.2024.1327405

**Published:** 2024-03-27

**Authors:** Robert Schauner, Jordan Cress, Changjin Hong, David Wald, Parameswaran Ramakrishnan

**Affiliations:** ^1^ Department of Pathology, Case Western Reserve University, Cleveland, OH, United States; ^2^ Department of Artificial Intelligence and Informatics, Mayo Clinic, Jacksonville, FL, United States; ^3^ The Case Comprehensive Cancer Center, Case Western Reserve University, Cleveland, OH, United States; ^4^ Department of Pathology, University Hospitals Cleveland Medical Center, Cleveland, OH, United States; ^5^ Department of Pathology, Louis Stokes Cleveland VA Medical Center, Cleveland, OH, United States

**Keywords:** hexosamine biosynthetic pathway, O-GlcNAcylation, AML, OGT, OGA, leukemic stem cells, NF-κB, single cell RNA sequencing

## Abstract

**Introduction:**

Acute myeloid leukemia (AML) is the most common acute leukemia in adults with an overall poor prognosis and high relapse rate. Multiple factors including genetic abnormalities, differentiation defects and altered cellular metabolism contribute to AML development and progression. Though the roles of oxidative phosphorylation and glycolysis are defined in AML, the role of the hexosamine biosynthetic pathway (HBP), which regulates the O-GlcNAcylation of cytoplasmic and nuclear proteins, remains poorly defined.

**Methods:**

We studied the expression of the key enzymes involved in the HBP in AML blasts and stem cells by RNA sequencing at the single-cell and bulk level. We performed flow cytometry to study OGT protein expression and global O-GlcNAcylation. We studied the functional effects of inhibiting O-GlcNAcylation on transcriptional activation in AML cells by Western blotting and real time PCR and on cell cycle by flow cytometry.

**Results:**

We found higher expression levels of the key enzymes in the HBP in AML as compared to healthy donors in whole blood. We observed elevated O-GlcNAc Transferase (OGT) and O-GlcNAcase (OGA) expression in AML stem and bulk cells as compared to normal hematopoietic stem and progenitor cells (HSPCs). We also found that both AML bulk cells and stem cells show significantly enhanced OGT protein expression and global O-GlcNAcylation as compared to normal HSPCs, validating our in silico findings. Gene set analysis showed substantial enrichment of the NF-κB pathway in AML cells expressing high OGT levels. Inhibition of O-GlcNAcylation decreased NF-κB nuclear translocation and the expression of selected NF-κB-dependent genes controlling cell cycle. It also blocked cell cycle progression suggesting a link between enhanced O-GlcNAcylation and NF-κB activation in AML cell survival and proliferation.

**Discussion:**

Our study suggests the HBP may prove a potential target, alone or in combination with other therapeutic approaches, to impact both AML blasts and stem cells. Moreover, as insufficient targeting of AML stem cells by traditional chemotherapy is thought to lead to relapse, blocking HBP and O-GlcNAcylation in AML stem cells may represent a novel promising target to control relapse.

## 1 Introduction

Acute myeloid leukemia (AML) is the most common acute leukemia among adults. It has an overall poor prognosis, high relapse rate and its incidence increases with age (1, 2). Therapeutic progress for AML for the past 4 decades has been limited and an AML cure remains a major challenge with the existing treatment modalities. To date, most AML patients still rely on traditional chemotherapy and allogeneic bone marrow transplantation, which show the need to discover novel pathways and mechanisms involved in AML to develop new treatment strategies (3, 4). There have been several new agents approved in recent years to treat AML such as IDH (5, 6), and Fms-Like Tyrosine kinase 3 (FLT3) (7) inhibitors. However, these drugs have led to only modest improvements in patient survival and are only useful for subsets of patients with the relevant genetic abnormalities (8).

Targeting cell metabolism is emerging as a promising avenue for cancer therapy (9). For example, inhibitors of the metabolic enzyme IDH have been approved for use in AML patients with IDH mutations (6). Previous studies have shown AML cells are adaptable to diverse metabolic pathways and use fatty acids and amino acids to enable mitochondrial metabolism (10). We have previously shown drug resistant AML stem cells (LSCs) prioritize oxidative metabolism over glycolysis and LSCs also increase their dependence on fatty acids and amino acids during disease progression (11). Thus, differential utilization of metabolic pathways has been reported to play a role in specific AML cell subsets and represents a unique vulnerability for targeting cancer cells as opposed to normal cells.

**Figure 1:**
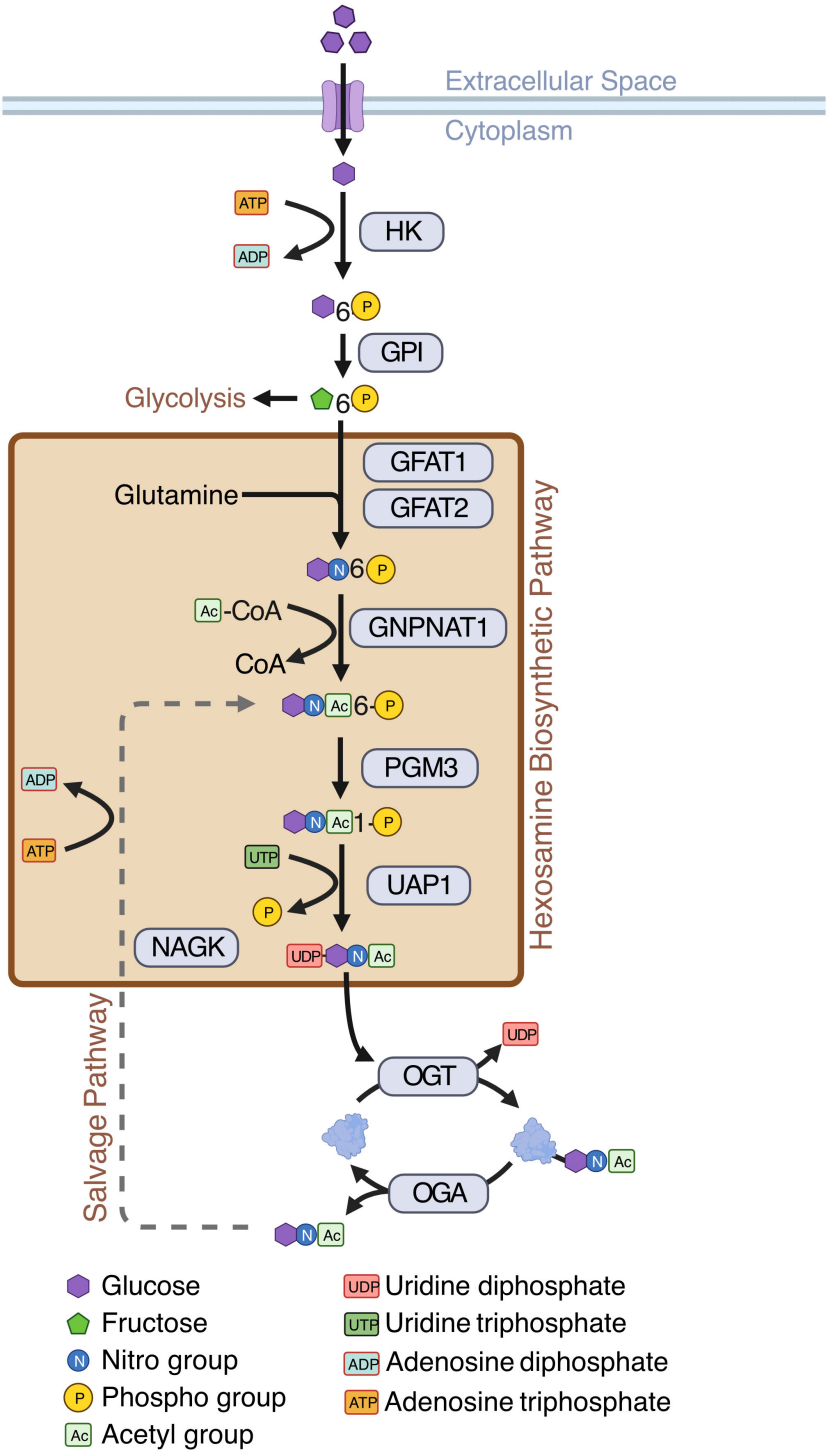
Hexosamine Biosynthetic Pathway (HBP). Schematic showing enzymes and substrates involved in *de novo* and salvage HBP. Created with BioRender.

In general, cancer cells, including AML cells, depend on anaerobic glycolysis which is known as the Warburg effect (12)—a less efficient way of energy production compared to mitochondrial oxidative phosphorylation. This results in increased uptake of glucose by cancer cells for their increased energy need and proliferation (13). Cancer cells also consume large amounts of glutamine, a precursor amino acid for the synthesis of glucosamine. Increased glucose flux and glutamine consumption has been shown to act as prominent initiators of the hexosamine biosynthetic pathway (HBP), which is a minor arm of glucose metabolism (14). The HBP usually accounts for a minor fraction of total glucose metabolism, with its end product, UDP-GlcNAc, acting as the substrate for a post-translational modification (PTM) called O-GlcNAcylation (14)–a reversible, dynamic, covalent modification analogous to phosphorylation. Just a single pair of enzymes, O-GlcNAc transferase (OGT) and O-GlcNAcase (OGA) mediates O-GlcNAcylation of all cellular proteins. OGT transfers GlcNAc from UDP-GlcNAc to target proteins and OGA removes O-GlcNAc from modified proteins (14) (**Figure 1**). O-GlcNAcylation occurs primarily at serine and threonine residues of intracellular proteins, and it often competes with or alters phosphorylation (15). O-GlcNAcylation regulates various cellular processes including transcription, cell signaling, metabolism, cell cycle, cell survival, stress, and oncogenesis (15, 16).

Increased O-GlcNAcylation is implicated in both hematopoietic and solid cancers where it controls cell proliferation and metastasis (17, 18). O-GlcNAcylation is a key player in hematological malignancies such as chronic lymphocytic leukemia (CLL), pre-B-cell acute lymphocytic leukemia (preB ALL) and AML (19). Specific O-GlcNAcylation of signal transducer and activator of transcription 5 (STAT5) at threonine 92 has been reported both in CLL and AML cells. STAT5 O-GlcNAcylation at T92 enhances its tyrosine phosphorylation and promotes neoplastic proliferation of myeloid cells (20). Overall, it appears suppressing O-GlcNAcylation limits oncogenesis, and an increase in O-GlcNAcylation supports oncogenesis (21). Previously, it has been shown global O-GlcNAcylation is increased in AML cell lines and primary bulk cells and HBP inhibition resulted in AML cell apoptosis, sparing normal peripheral blood mononuclear cells (PBMC) (22), but a direct comparison of O-GlcNAcylation in AML and HSPC was lacking. Interestingly, moderate HBP or O-GlcNAcylation inhibition induced AML cell differentiation (22). These data suggest the HBP and O-GlcNAcylation play a role in bulk AML cell survival and differentiation arrest. Another study found there is a positive correlation between O-GlcNAcylation levels and AML chemoresistance. Combination treatment of the OGT inhibitor, OSMI-1, with doxorubicin resulted in a synergistic increase in apoptosis of AML cells (23).

As AML is a heterogeneous disease with a wide range of genetic subtypes, it is important to understand the role of the HBP in AML across patients (24). Likewise, it is important to appreciate the HBP at both the AML blast stage as well as in LSCs, which are crucial for AML initiation. Because LSCs are typically chemoresistant, it is particularly important to study HBP enzyme expression patterns in this population. To elucidate the biology of AML and to gain further understanding of O-GlcNAcylation dysregulation in AML, we analyzed single-cell and bulk RNA-sequencing data to evaluate the expression of HBP enzymes in AML patient’s blasts and LSCs in comparison to healthy controls. This study contributes to a better understanding of the regulation of the HBP and O-GlcNAcylation in AML blasts and LSCs. It also provides insight into the functional consequences of enhanced O-GlcNAcylation by studying its role in promoting NF-κB signaling. This implicates the HBP as a relevant metabolic pathway that may be targeted to develop prognostic biomarkers as well as improve AML therapeutics.

## 2 Methods

### 2.1 Cell culture conditions

OCI-AML3 cells (DSMZ) were cultured in RPMI-1640 media with 10% SCS, 100 U/mL penicillin/streptomycin, and 1% L-glutamine. Cells were incubated at 37°C with 5% CO_2_.

### 2.2 Western blotting for global O-GlcNAcylation

OCI-AML3 cells (5 x 10^5^ cells/mL) were cultured with 25 µM Thiamet-G (Cayman Chemicals, Cat# 1009816-48-1), 25 µM OSMI-1 (MedChem Express, Cat # HY-119738), or vehicle control (DMSO) for 16 hours. Cells were lysed for 15 minutes on ice with Triton lysis buffer (1% Triton-X100, 20 mM HEPES [pH 7.6], 0.1% SDS, 0.5% Sodium deoxycholate, 150 mM NaCl, 1 mM EDTA) supplemented with protease inhibitor cocktail (ThermoFisher, Cat# A32955). Cell lysates were resolved through 7% SDS-PAGE gels. Proteins from the gel were transferred onto nitrocellulose membranes which were blocked with 5% Bovine Serum Albumin (prepared using Tris-buffered Saline containing 0.1% Tween). Membranes were probed with primary antibodies followed by HRP-conjugated secondary antibodies. The blots were developed with enhanced chemiluminescence substrate (GenDepot) and exposed to X-ray films (Fujifilm). The following antibodies were used: O-GlcNAc (Santa Cruz, Cat# sc-59624), and β-tubulin (Santa Cruz, Cat# sc-9104).

### 2.3 Western blotting for cytoplasmic and nuclear NF-κB

OCI-AML3 cells (5 x 10^5^ cells/mL) were cultured with 25 µM OSMI-1 or vehicle control for 4 hours. For cytoplasmic and nuclear fractionation, cells were lysed with cytoplasmic lysis buffer (10 mM HEPES pH 7.6, 10 mM KCl, 0.1 mM EDTA, 0.1 mM EGTA) on ice for 15 minutes. NP-40 was added to lysates to a final concentration of 0.625% and vortexed for 10 seconds. Lysates were then centrifuged at 12,000 xg for 30 seconds at 4°C and supernatants were collected for the cytoplasmic fraction. Pellets were washed once with cytoplasmic lysis buffer and resuspended in nuclear lysis buffer (20 mM HEPES pH 7.6, 400 mM NaCl, 1 mM EDTA, 1 mM EGTA). Lysates were kept on ice for 30 minutes before being centrifuged at 12,000 xg for 10 minutes at 4°C. The supernatant was then collected for the nuclear fraction. Both lysis buffers were supplemented with protease inhibitor cocktail (ThermoFisher, Cat# A32955). Cell lysates were resolved through 7% SDS-PAGE gels. Proteins from the gel were transferred onto nitrocellulose membranes which were blocked with 5% Bovine Serum Albumin (prepared using Tris-buffered Saline containing 0.1% Tween). Membranes were probed with primary antibodies followed by HRP-conjugated secondary antibodies. The blots were developed with enhanced chemiluminescence substrate (GenDepot) and exposed to X-ray films (Fujifilm). The following antibodies were used: O-GlcNAc (Santa Cruz, Cat# sc-59624), β-tubulin (Santa Cruz, Cat# sc-9104), p65 (Santa Cruz, Cat# sc-372), p50 (Cell Signaling, Cat# 13586S), c-Rel (Cell signaling, Cat# 4727S), and Lamin A/C (Santa Cruz, Cat# sc-20681).

### 2.4 Quantitative real-time PCR

OCI-AML3 cells (5 x 10^5^ cells/mL) were cultured with 25 µM OSMI-1 or vehicle control for 4 hours. RNA was isolated from cells using EZ10 DNAaway RNA miniprep kit (BioBasic) and quantified with a NanoDrop spectrophotometer. cDNA was synthesized from 1 µg of RNA using the High Capacity cDNA Reverse Transcription Kit (Applied Biosystems). Quantitative real-time PCR was performed using HotStart™ 2X Green qPCR Master Mix (APExBIO). Gene expression values were normalized to the housekeeping gene RPL32 and fold changes were calculated using the ΔΔCt method. The following primers were used:

RPL32 F: AGCTCCCAAAAATAGACGCAC, R: TTCATAGCAGTAGGCACAAAG

c-Myc F: CCTGGTGCTCCATGAGGAGAC, R: CAGACTCTGACCTTTTGCCAGG

Cyclin D1 F: GCGGAGGAGAACAAACAGAT, R: TGAACTTCACATCTGTGGCA

Cyclin E1 F: CCCGGTCATCATCTTCTTTG, R: AGAAATGGCCAAAATCGACA

### 2.5 Primary AML and healthy donor cells

Peripheral blood and bone marrow samples from AML patients and healthy donors were obtained from the Case Western Reserve University Hematopoietic Biorepository and Cellular Therapy Core. The core performed Ficoll-density purification to isolate mononuclear cells (MNC) and cryopreserved them in liquid nitrogen. For healthy PBMCs, MNCs were isolated from healthy blood using Ficoll-Paque Premium density gradient media (Cytiva, Cat# 17544652) and Leucosep™ tubes (Grenier Bio-One, Cat# 227290). PBMCs were cryopreserved prior to processing for flow cytometry analysis.

### 2.6 Intracellular O-GlcNAcylation staining and flow cytometry

Patient samples were thawed, washed, and treated with 25 μg/ml of DNase I in 1% BSA/PBS to dissociate cell clumps. Cells were washed to remove DNase I and 2.5 x 10^6^ cells per sample were transferred to a well in a 96-well round bottom plate (Fisher, Cat# 12-565-65). Cell viability was assessed using Zombie NIR Fixable dye following manufacturer’s instructions (BioLegend, Cat# 423105). Cells were then blocked with TruStain Human FcX Blocking Buffer followed by incubation with antibodies against CD34 (BV421, BD Biosciences, Cat#745259) and CD38 (PE, BioLegend, Cat# 303506). After surface staining, cells were fixed and permeabilized using the True Nuclear Transcription Factor Buffer Set (BioLegend, Cat# 424401) and then stained with the RL2 antibody recognizing O-GlcNAcylated proteins (Invitrogen, Cat# 51-9793-42) and an anti-O-GlcNAc Transferase (OGT) antibody (Santa Cruz, Cat# sc-32921). For OGT staining, anti-rabbit IgG (AF488, Cat# A-11008) was used to detect presence of the anti-OGT antibody. Samples were then acquired using an Attune NxT acoustic focusing flow cytometer and analyzed using FlowJo V10. Fluorescence minus one (FMO) controls were used to set positive gates for RL2 and OGT positive cells. Median fluorescence intensities across groups were compared using a one-way ANOVA with Dunnett’s multiple comparison test for analyses with more than two groups or a Student’s t-test with analyses with two groups.

### 2.7 Cell cycle and proliferation analysis

OCI-AML3 (3 x 10^5^ cells/mL) cells were cultured with OSMI-1 (25 μM) or vehicle control for 2 days. Cells were permeabilized with 70% ethanol for 30 minutes at 4°C and stained with propidium iodide. To quantify cell numbers, acquisition settings were kept consistent across all samples. Cell cycle progression was measured using an Attune NxT acoustic focusing flow cytometer and analyzed using FlowJo V10.

### 2.8 Bioinformatic processing of single-cell sequencing data

For the analysis of data from Stetson et al. (11), RNA was normalized using SCTransform (25, 26). Differential expression was done using logistic regression with log fold change cutoffs reduced to 0. CCA integration between all samples was performed using Seurat v4 (27–30). HBP members were spiked into the list of integrated features to ensure their presence during integration. Clustering was performed using FindNeighbors and FindClusters with the default parameters and the 2000 most highly variable genes.

For the analysis of data from Van Galen et al. (31), RNA counts were CPM normalized and log-scaled. Cell types were used as previously defined (31). GMP, HSPC and Progenitor cells were grouped into a HSPC group (Healthy Donors) or LSC-like group (AML patients), and remaining malignant cells were labeled AML Blasts. Differential expression was done using logistic regression with log fold change cutoffs reduced to 0 on the imputed values.

### 2.9 Analysis of TCGA, BeatAML, TARGET, St. Jude, and GTEx data

Samples were imported into R and categorized by age (Adult > 29) for TCGA, BeatAML, TARGET, and data from St. Jude Children’s Research Hospital’s St. Jude Cloud (SJC-DS-1013 and SJC-DS-1009) cohorts. Whole blood samples from GTEx were used as a normal comparison along with normal cells from GSE198919. Genes not found across all datasets were removed. Counts were normalized using voom from limma (32–34) v3.54.2. GSVA (35) v1.46.0 was used to generate enrichment scores for gene ontology and hallmark gene sets. Cutoffs for OGA and OGT were determined using the 10% and 90% quantile across all samples. Differential expression was conducted using eBayes and topTable for both RNA counts and GSVA enrichment scores (32, 33).

### 2.10 Statistics

Differential expression was conducted using limma-eBayes (32–34) for bulk RNA-sequencing data and Seurat FindMarkers with method=“LR” and fold change cutoffs set to 0 (25, 28, 30, 36) for single-cell RNA-sequencing data. For flow cytometric analyses, median florescence intensity values were compared using a one-way ANOVA with Dunnett’s multiple comparison test for analyses with more than two groups or a Student’s t-test for analyses with two groups.

## 3 Results

### 3.1 OGA and OGT expression is heterogeneous in LSCs

**Figure 2:**
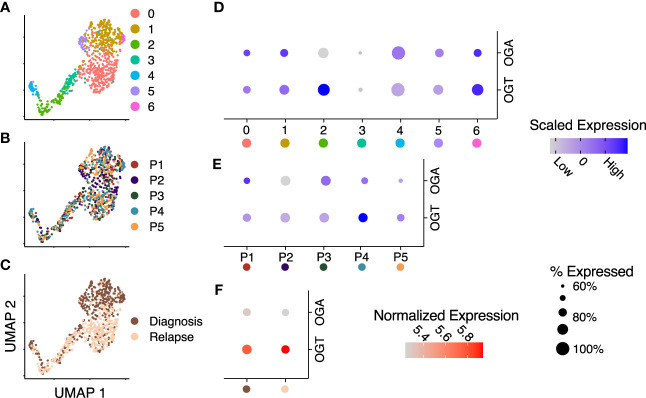
OGT expression is increased across several RNA-sequencing datasets. **(A–C)** Integrated UMAP embeddings of clusters **(A)**, patients **(B)** and timepoints **(C)**. **(D, E)** Dot plot with scaled average expression of OGA and OGT per cluster **(D)** and patient **(E)**. **(F)** Dot plot with log-normalized expression of OGA and OGT per timepoint. **(D–F)** Size of dots represents the percentage of cells with more than one UMI count for OGT or OGA, respectively. Data is from Stetson LC, et al (11). Data includes 721 cells from 5 patients.

Bulk AML cells show enhanced HBP activity and O-GlcNAcylation compared t0 PBMCs and chemotherapeutic drugs have been shown to further enhance O-GlcNAcylation in AML cells (19, 22, 23). In this manuscript, we use bulk cells to refer to MNC from blood or bone marrow in AML patients and AML blasts to refer to non-LSC AML cells. A comparison of O-GlcNAcylation in AML to their comparable healthy counterpart myeloid progenitors has not been done previously. Recently, it was also shown that inhibition of O-GlcNAcylation promotes the differentiation of LSCs (37). Previous single-cell studies analyzing primary AML samples demonstrate it is a highly heterogeneous disease both within a patient and between patients (38). The metabolic pathways, oxidative phosphorylation and glycolysis, also show remarkable gradience among patients and cell types of individual patients, however, the role of O-GlcNAc cycling enzymes at the single-cell level remains unknown. To study whether patient and cell type specific heterogeneity exists in these enzymes at the single-cell level in leukemic stem cells (LSCs), we integrated single-cell RNA-sequencing data on LSCs from serial diagnostic and relapse samples from 5 AML patients (11). Uniform manifold approximation and projection (UMAP) visualization of shared-nearest neighbor clustering of all the cells from these patients found 7 unique clusters (**Figure 2A**) with intermixed expression of *OGT* and *OGA* between patients (**Figure 2B**) and timepoints, i.e., diagnosis and relapse (**Figure 2C**). Differential expression analysis of *OGT* and *OGA* (**Figure 2D**, **Supplementary Table 1**) showed clusters with lower expression levels with less cells expressing *OGA* and *OGT* (cluster 3) as well as higher expression levels of *OGT* and *OGA* in a higher proportion of cells (cluster 6). Cluster 2 shows higher *OGT* expression without higher *OGA* expression, suggesting cells in this cluster are skewed towards higher levels of O-GlcNAcylation (**Figure 2D**). We observed no difference (p = 1.0) in *OGT* and *OGA* expression between patients (**Figure 2E**). Interestingly, the expression of *OGT* and *OGA* were unchanged between diagnosis and relapse samples (**Figure 2F**). These differences in the expression of *OGT* and *OGA* suggest cycling of O-GlcNAcylation may be different in LSC subsets/clusters that may affect their function.

### 3.2 Expression of OGT and classical HBP enzymes are increased in AML blasts and LSCs

**Figure 3:**
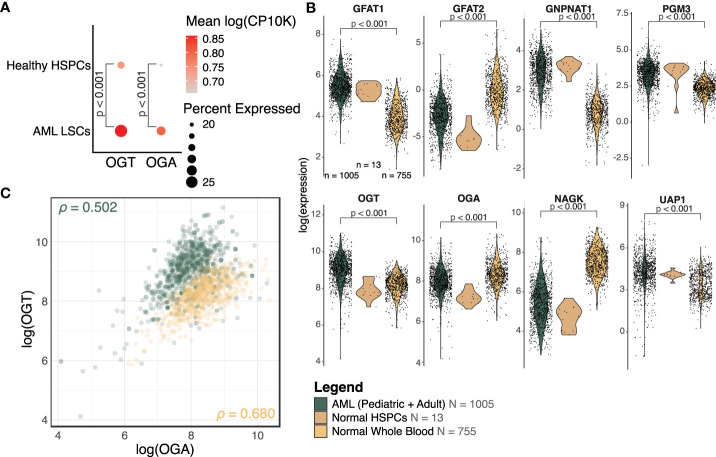
OGT and many HBP members are increased in AML. **(A)** Dot plot of OGT and OGA expression in LSCs and HSPCs from healthy donors (HD). The scRNA-Seq data is from van Galen, P et al. (31). **(B)** Expression levels of HBP members from bulk RNA-sequencing. Expression has been normalized using voom. AML: n = 1005, Normal HSPC: n = 13, Normal Whole Blood: n = 755, also shown in the legend. DE analysis results are shown where p < 0.05. **(C)** Scatterplot of OGT and OGA expression values in AML (green) and normal (orange) samples. ρ = Pearson correlation between OGA and OGT expression. **(B, C)** Data is from TCGA, TARGET, BeatAML, St. Jude Children’s Research Hospital, GTEx, and GSE198919.

To assess differences in the expression of enzymes involved in the HBP between healthy and AML cells, we collected publicly available single-cell RNA-sequencing data on AML and healthy donor bone marrow (31). This study used a combination of single-cell sequencing and single-cell genotyping to classify cells into non-malignant or AML cells, based on known AML mutations. The AML cells were separated into blasts and LSCs. We compared *OGT* and *OGA* expression between LSCs and HSPCs. We found LSCs had a higher expression of *OGT* compared to HSPCs (p < 0.001, logFC = 0.17, **Figure 3A**). We also found an increase in *OGA* expression in LSCs compared to HSPCs (p < 0.001, logFC = 0.20, **Figure 3A**), although overall *OGA* expression was lower than *OGT*.

We also analyzed the expression levels of HBP enzymes which control UDP-GlcNAc generation using bulk RNA-sequencing data across several cohorts (TCGA, BeatAML, TARGET, and St. Jude Children’s Research Hospital). We found HBP enzymes involved in *de novo* UDP-GlcNAc generation such as *GFPT1* (transcribing GFAT1, logFC = 1.43, p < 0.001), *GNPNAT1* (logFC = 2.06, p < 0.001), *PGM3* (logFC = 0.90, p < 0.001), and *UAP1* (logFC = 0.83, p < 0.001) were upregulated in AML patients as compared to healthy donors, suggesting elevated HBP activity (**Figure 3B**, **Supplementary Table 2**). We also saw *OGT* expression was higher in samples from AML patients (logFC = 0.78, p < 0.001), which mirrored the same trend observed in the single-cell sequencing analysis (**Figure 3A**). On the other hand, AML patients had a moderately lower expression of OGA (logFC = -0.40, p < 0.001) as compared to healthy donors. However, the fold change observed was very small, warranting further studies on OGA expression at RNA and protein levels in AML to understand its biological significance and correlate it with altered O-GlcNAcylation. We also included normal HSPCs (n = 13) as a reference for the expression of HBP proteins in healthy hematopoietic progenitors—the cell type from which AML is thought to originate. Due to the low sample size making the comparison between normal HSPCs and AML cells underpowered, it is not possible to make any definitive conclusions on changes in the expression of HBP proteins among these groups.

While most HBP enzymes had increased expression in AML, *NAGK* expression was lower in AML than the healthy controls (**Figure 3B**, logFC = -2.10, p < 0.001). *NAGK* is the key enzyme involved in the salvage pathway (39), where it interacts with free N-Acetylglucosamine removed from previously O-GlcNAcylated proteins and converts it to N-Acetylglucosamine-6-Phosphate allowing it to be recycled to produce UDP-GlcNAc (**Figure 1**). Downregulation of this enzyme could indicate AML cells are less dependent on the salvage pathway to generate UDP-GlcNAc, and instead rely more on *de novo* synthesis.

We also found the expression of *GFPT2* (transcribing GFAT2) was lower (logFC = -2.73, p < 0.001) in AML samples as compared to normal controls (**Figure 3B**, **Supplementary Table 2**). GFAT2 is one of the two proteins (GFAT1 and GFAT2) which catalyze the rate-limiting step of the HBP which converts fructose-6-phosphate to glucosamine-6-phosphate (40) (**Figure 1**). This event is the important branch that directs sugar to the HBP instead of glycolysis (**Figure 1**). Downregulation of GFAT2 and upregulation of its homologue, GFAT1, suggest AML cells may preferentially rely on GFAT1 for HBP activation. We observed small differences in *OGT* (logFC = 0.16, p = 0.002), *PGM3* (logFC = 0.34, p < 0.001), and *GFPT1* (logFC = 0.10, p = 0.019), between pediatric and adult AML samples, but no difference in any other HBP enzymes (**Supplementary Table 2**). Though there are slight differences in HBP enzyme expression between adult and pediatric AML patients, these differences are much smaller than the changes between AML (pediatric and adult) and normal controls (**Figure 3B**).

We also assessed the relationship between *OGT* and *OGA* expression. The Pearson correlation coefficient in AML patients (ρ = 0.502, 95% CI = 0.454 - 0.547) and normal controls (ρ = 0.680, 95% CI = 0.640 - 0.717, **Figure 3C**), revealed a substantial correlation between these two genes. This finding supports the idea that flux through the HBP is regulated concurrently by co-regulation of these genes. We also found greater OGT expression and a higher OGT : OGA ratio in AML cells (green) compared to normal controls (orange) which would contribute to higher levels of protein O-GlcNAcylation. Overall, this expression analysis shows AML cells have an increased expression of OGT including in the LSCs as compared to HSPCs and PBMCs.

### 3.3 Patients with high levels of OGT and OGA show distinct gene set enrichment

**Figure 4:**
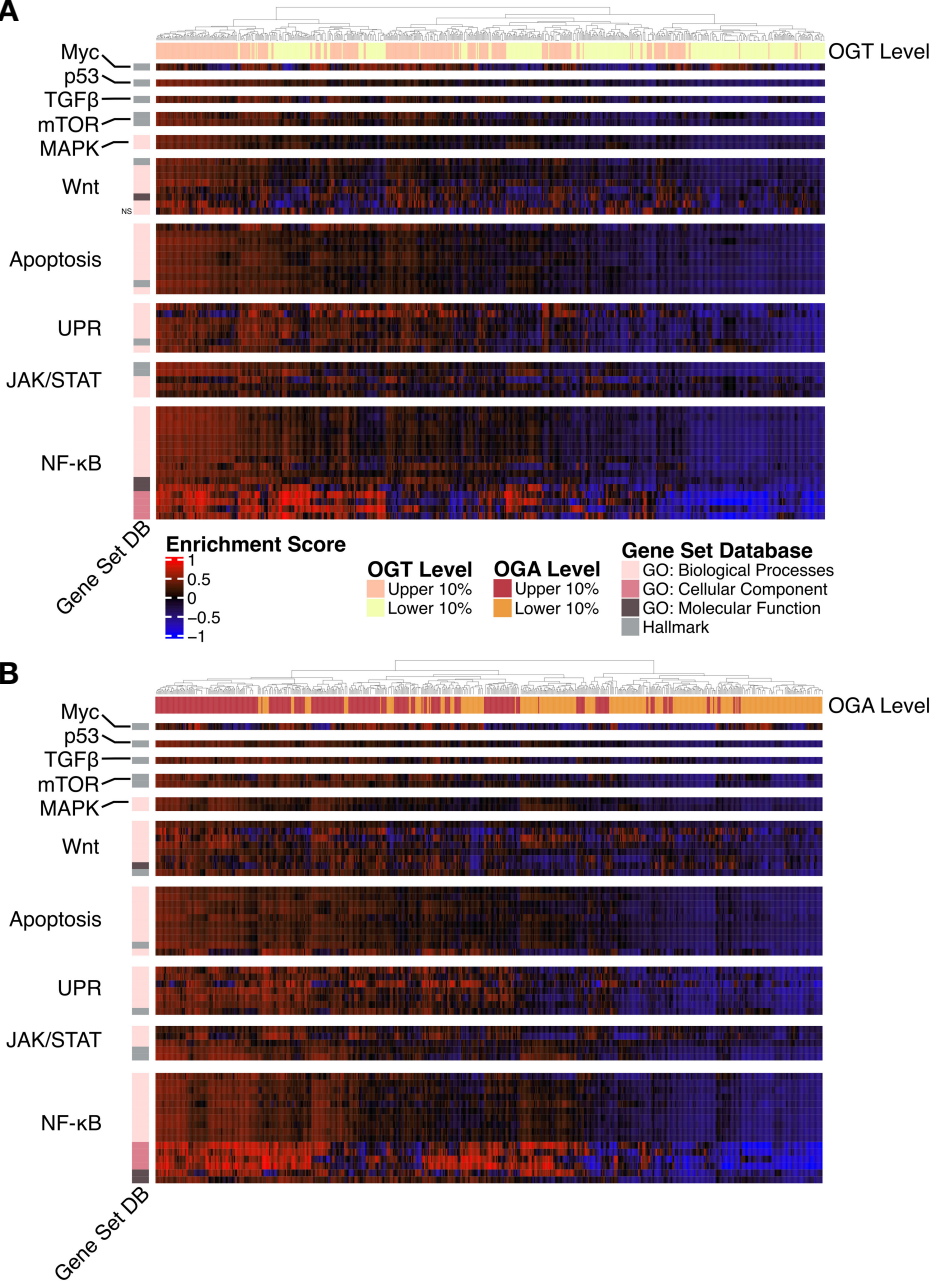
Gene Ontology and Hallmark GSVA. Heatmap of the GSVA enrichment score per sample from AML samples. All gene sets pass a p-value threshold of 0.05 unless otherwise noted with NS based on a DE analysis. Gene sets were manually annotated with a category. n = 636. **(A)** Upper and lower 10% of samples by OGT expression. **(B)** Upper and lower 10% of samples by OGA expression.

A majority of the known O-GlcNAcylated proteins are transcription factors and changes in O-GlcNAcylation significantly alter gene expression in several disease states (41, 42). To study the effect of changes in O-GlcNAcylation on gene expression in AML, we performed a gene set variance analysis using 636 samples with the highest (n = 318) and lowest (n = 318) *OGT* or *OGA* expression. We selected the top 10% and bottom 10% of samples based on *OGT* (**Figure 4A**, **Supplementary Table 3**) and *OGA* (**Figure 4B**, **Supplementary Table 4**) expression. We found samples expressing high levels of *OGT* showed higher enrichment in multiple gene sets related to proliferation including PI3K/AKT/mTOR, JAK/STAT, Wnt/β-catenin, and NF-κB signaling (**Figure 4A**). Interestingly, we also observed similar trends in samples expressing high levels of OGA (**Figure 4B**), likely resulting from the ability of OGT and OGA to transcriptionally regulate each other (43). Thus, as OGT expression increases, OGA expression is also expected to increase. This suggests that O-GlcNAc cycling is important in promoting cell proliferation pathways, as shown previously in several cancer types where OGA and OGT were both upregulated (44).

O-GlcNAcylation has been shown to influence activation of many of these pathways by modifying multiple proteins involved in these signaling cascades (19). Specifically, the up-regulation of gene sets involved in NF-κB signaling, such as the formation of the NF-κB p65/p50 complex as well as downregulation of those involved in the inhibitor of κB (IκB) and NF-κB complex. O-GlcNAcylation levels have been positively correlated with NF-κB activity across multiple cancer types (45). NF-κB p65 O-GlcNAcylation reduces affinity to IκB allowing for nuclear translocation (46). In addition, O-GlcNAcylation of NF-κB has also been shown to regulate its binding to certain promoter regions (47, 48). Thus, the specific enrichment of the NF-κB pathway in the gene set analysis emphasizes a prominent correlation with OGT/OGA expression and NF-κB activation, which might regulate AML survival, proliferation, and the evasion of apoptosis.

Similarly, we also found increased expression of *OGT* and *OGA* increases enrichment of the Hallmark c-Myc targets gene set (**Figures 4A, B**). This is consistent with previous findings showing c-Myc O-GlcNAcylation promotes its stability by inhibiting ubiquitination (49). c-Myc regulates genes involved in proliferation, survival, and metabolism. Importantly, c-Myc promotes glutamine metabolism and controls GLUT-1 expression (50), thus participating in a positive feedback loop to further increase HBP activity through increased glucose uptake. Justifying the enrichment of AKT/mTOR pathway in the gene set, O-GlcNAcylation stabilizes transcriptional co-activators such as DDX5 and TCL1, which play a role in regulating AKT expression and subsequent mTOR activation (51, 52).

We also found higher levels of *OGA* and *OGT* were associated increased enrichment of unfolded protein response (UPR) related GO terms suggesting a link between O-GlcNAcylation and endoplasmic reticulum stress in AML. Increase in *OGT*/*OGA* expression and O-GlcNAcylation could provide a protective role for AML cells as increased O-GlcNAcylation abrogates the pro-apoptotic arm of the UPR (53).

### 3.4 AML blasts and LSCs show enhanced protein O-GlcNAcylation and OGT expression

**Figure 5:**
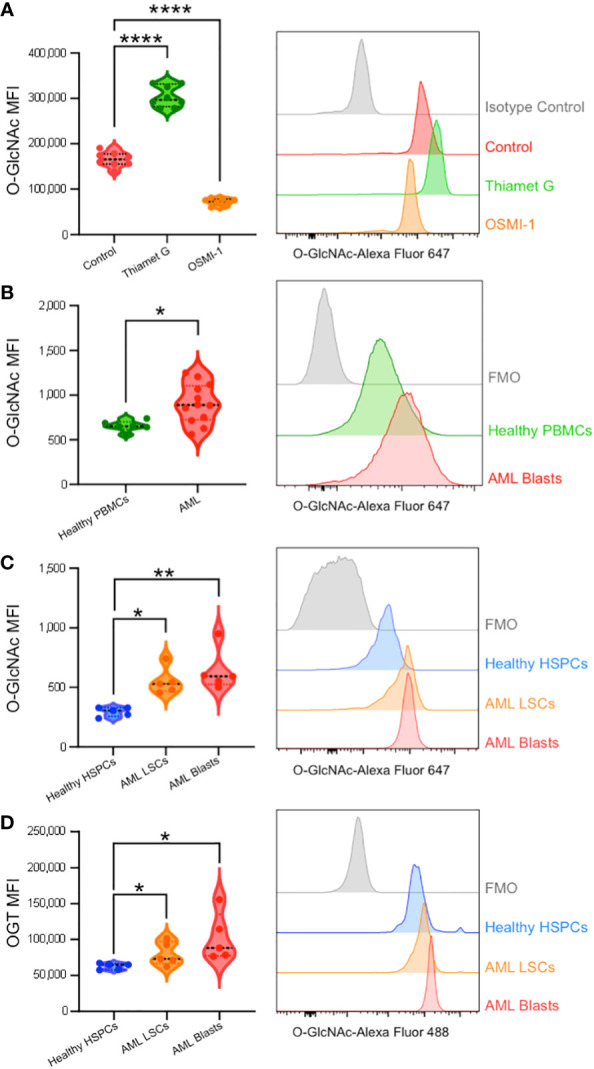
Intracellular flow cytometry confirms AML patients display enhanced O-GlcNAcylation and OGT expression. **(A)** O-GlcNAc staining of OCI-AML3 cells by flow cytometry with vehicle control, Thiamet G (25 μM), or OSMI-1 (25 μM). n = 8 **(B)** Intracellular O-GlcNAc levels from healthy donor derived PBMCs (Healthy PBMCs, n = 6) and CD34+CD38+ AML cells from PBMCs or BM-MNCs (AML, n = 12). **(C, D)** O-GlcNAc levels **(C)** and OGT protein expression **(D)** in BM-MNCs isolated from healthy donors (n = 5) or AML patients (n = 5). HSPC: CD34+ from healthy donor, LSCs: CD34+CD38dim, AML Blasts: CD34+CD38+. **(A–D)** Representative histograms are shown on the right. Statistical analysis was done using a one-way ANOVA with Dunnett’s multiple comparison test **(A, C, D)** or a Student’s t-test **(B)**. *, P < 0.05; **, P < 0.005; ****, P < 0.0001. **(B–D)** FMO – Fluorescence minus one controls.

To validate our single-cell RNA-sequencing and bulk RNA analyses findings and show the upregulation of HBP enzymes is reflected at the protein level, we performed flow cytometry to analyze cellular O-GlcNAcylation and OGT protein expression. First, we optimized intracellular O-GlcNAcylation staining and detection by flow cytometry using OCI-AML3 cells and found reliable and reproducible O-GlcNAcylation enhanced with Thiamet G (OGA inhibitor) and diminished with OSMI-1 (OGT inhibitor) (**Figure 5A**). Western blot analysis was also used to confirm O-GlcNAcylation levels were seen through flow cytometry (**Supplementary Figure 2A**). Next, we studied AML blasts and LSCs from AML patient bone marrow and HSPCs as well as PBMCs from healthy donors (**Table 1**). Consistent with our RNA expression analyses, we found AML blasts showed higher levels of O-GlcNAcylation compared to healthy PBMCs (**Figure 5B**, **Supplementary Figure 2B**). However, since PBMCs mainly consist of differentiated hematopoietic cells, many of which are lymphocytes, they do not represent the optimal comparison to AML cells.

**Table 1:** Patient Demographics for flow cytometric validation.

Patient #	Age	Sex	AML subtype	Recurrent Genetic Abnormalities	Cell Source
1	41	F	M4	FLT3-ITD, NPM1c, c-Kit	BM
2	61	M	M4	FLT3-ITD, NPM1c	BM
3	33	M	M3	PML/RARA	BM
4	55	M	AML-MRC	NPM1c, CEBPA	BM
5	70	M	AML-MRC	NRAS, SF3B1, ETV6	BM
6	65	M	M2	None were identified	BM
7	73	M	M2	Tet2	BM
8	42	M	M2	FLT3-ITD, c-Kit	BM
9	33	F	M5	None were identified	BM
10	84	F	M1	IDH1	BM
11	51	M	M0	None were identified	BM
12	49	M	M2	FLT3-ITD, c-Kit	PB
13	71	M	M4	None were identified	BM
14	76	M	AML-MRC	None were identified	BM
15	74	M	M4	NPM1c	PB
16	14	M	M4	None were identified	BM
17	NA	NA	NA	NA	BM

AML-MRC, AML with myelodysplasia related changes; BM, Bone marrow; PB, Peripheral Blood; NA, Data not available.

**Figure 6:**
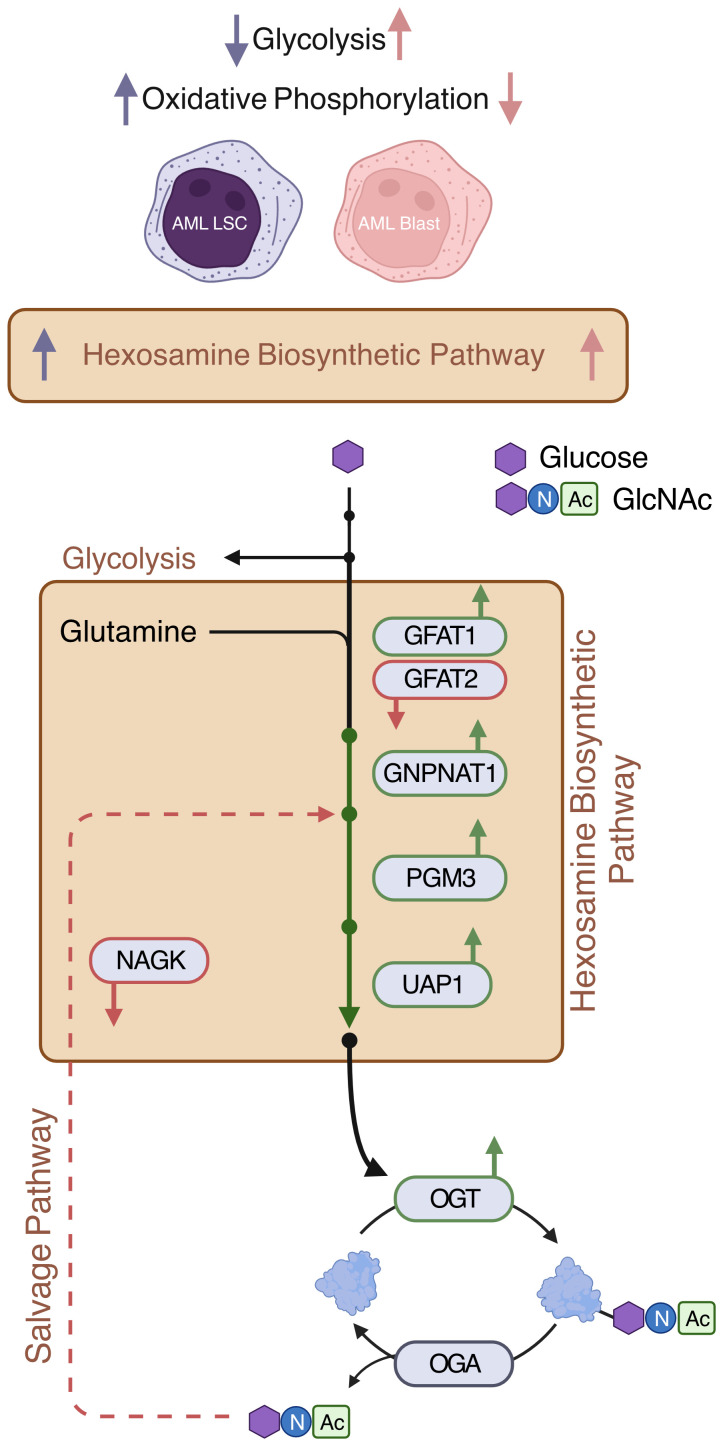
Expressions of de novo HBP enzymes are increased in AML. AML LSCs show increased oxidative phosphorylation and AML blasts show increased glycolysis (11). This study shows that HBP is increased both in AML LSCs and AML blasts. De novo HBP in AML may utilize the rate limiting step enzyme GFAT1. OGT is elevated in AML. Expression of salvage HBP enzyme NAGK is decreased in AML Created with BioRender.

To better compare AML cells to their non-malignant counterparts, we obtained control bone marrow samples without the presence of malignant cells and HSPCs were identified based on CD34 expression (**Supplementary Figure 1A**). AML bone marrow was stained with CD34 and CD38 antibodies to subcategorize into LSCs (CD34^+^CD38^-^) and Blasts (CD34^+^CD38^+^) (**Supplementary Figure 1B**). We found AML blasts and LSCs showed higher levels of O-GlcNAcylation as compared to HSPCs from healthy individuals with normal bone marrow (**Figure 5C**). In addition, we observed a 16.3% increase in total O-GlcNAcylation in between AML LSCs and blasts (p = 0.51). In line with increased O-GlcNAcylation, we observed AML blasts and LSCs both had higher OGT levels than HSPCs from normal bone marrow (**Figure 5D**). Further the expression pattern between AML LSCs and blasts followed the same trend as O-GlcNAcylation levels, with blasts having a 28.2% increase in OGT expression compared to the LSCs (p = 0.26) (**Figure 5D**). Indeed, we found OGT protein expression and cellular O-GlcNAcylation levels were correlated via a Pearson’s correlation with 2000 bootstrap replicates (ρ = 0.376, 95% CI = 0.368 - 0.384, **Supplementary Figure 2C**, **Supplementary Table 5**). This suggests hyper-O-GlcNAcylation seen in AML is partly regulated through OGT expression and not just increased uptake of glucose and glutamine (**Figure 6**).

### 3.5 Inhibiting O-GlcNAcylation decreases NF-κB activity and limits cell cycle progression in OCI-AML3 cells

**Figure 7:**
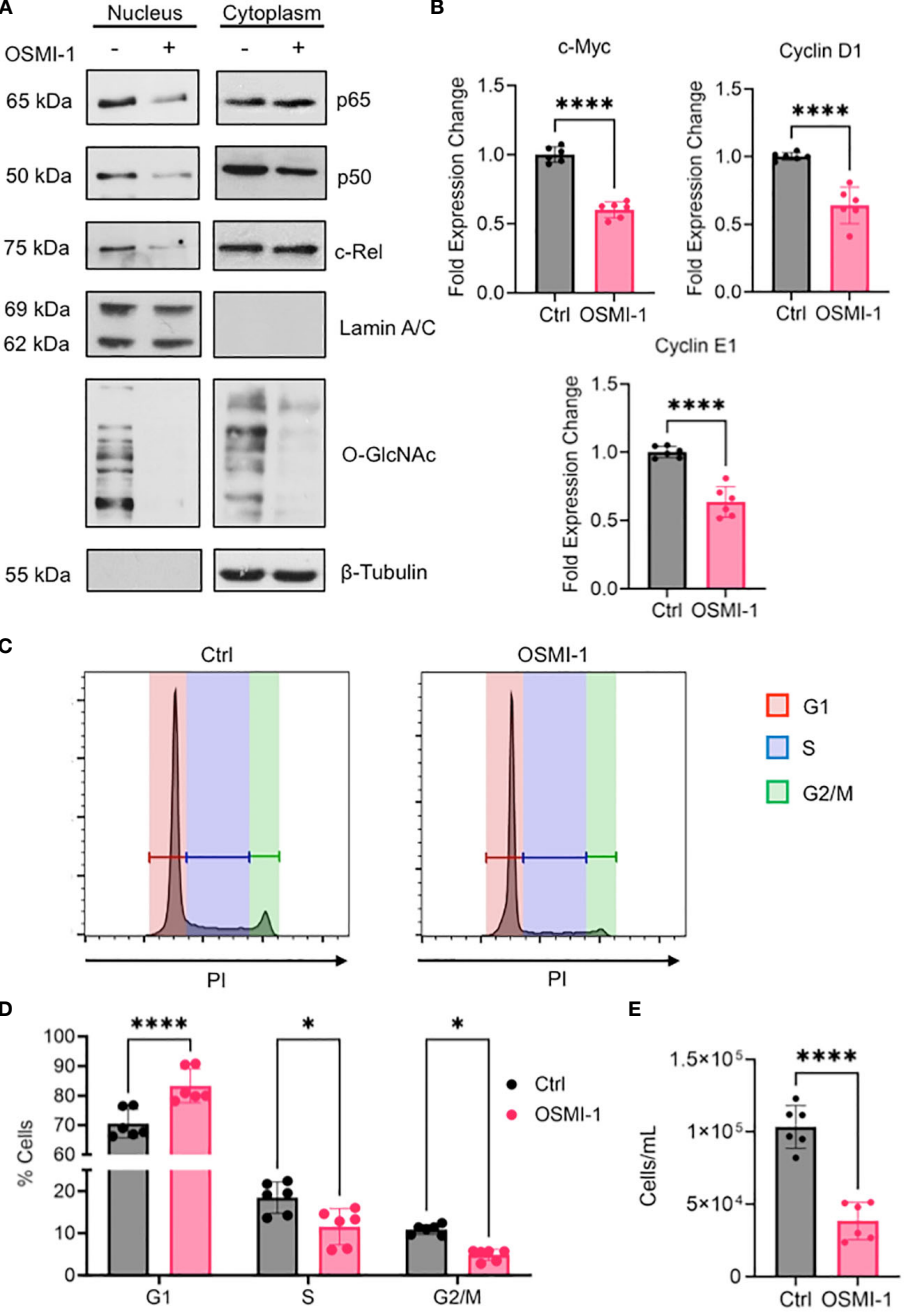
**(A)** OCI-AML3 cells were cultured in the presence or absence of OSMI-1 (25 μM) for 4 hours. Cytoplasmic and nuclear extracts were immunoblotted for indicated NF-κB subunits. **(B)** Gene expression of c-Myc, Cyclin D1, and Cyclin E1 in OCI-AML3 cells after 4 hours of OSMI-1 (25 μM) treatment measured by quantitative real-time PCR. Data shows 3 biological replicates performed with technical duplicates (n = 6). **(C)** OCI-AML3 cells were treated with OSMI-1 (25 μM) for 48 hours prior to permeabilization and staining with propidium iodide (PI). Representative histograms showing proportion of cells in each phase of the cell cycle: Red=G1, Blue=S, and Green= G2/M. **(D)** Bar graph showing cell cycle phases representing 3 individual experiments performed with technical duplicates (n = 6). **(E)** Quantification of cell numbers using flow cytometry after 48 hours of OSMI-1 (25 μM) treatment (n=6). *, P < 0.05; ****, P < 0.0001.

The enrichment of gene sets involved in pro-proliferative signaling pathways in AML cells expressing high amounts of OGT and OGA (**Figures 4A, B**), suggests that proteins in these pathways are altered by O-GlcNAcylation to promote AML progression. As the most prominent enrichment was observed in the NF-κB pathway, we examined the effect of O-GlcNAcylation inhibition on NF-κB activity. NF-κB is commonly dysregulated in AML patients and AML cell lines resulting in constitutive activation of this pathway (54). NF-κB transcription factors are sequestered in the cytoplasm by Inhibitor of kappa-B proteins (IκBs). Their activation results from the degradation of IκB proteins, freeing NF-κB to allow their translocation to the nucleus and binding to the target gene promoters (55) To study the role of O-GlcNAcylation on NF-κB signaling in AML, we inhibited O-GlcNAcylation in OCI-AML3 cells that show constitutive NF-κB activity (56). We treated OCI-AML3 cells with the OGT inhibitor OSMI-1 (57) and analyzed NF-κB nuclear translocation. We inhibited O-GlcNAcylation in OCI-AML3 cells with OSMI-1 and analyzed levels of NF-κB subunits in the nucleus and cytoplasm. We found that canonical NF-κB subunits: p65, c-Rel, and p50 were present in the nucleus in basal conditions indicative of constitutive NF-κB activity. The nuclear levels of these NF-κB subunits decreased after OSMI-1 treatment (**Figure 7A**). Cytoplasmic expression of these subunits appeared unchanged suggesting that inhibiting O-GlcNAcylation impairs their ability to translocate to the nucleus. Furthermore, OSMI-1 treatment also inhibited the expression of NF-κB target genes c-Myc, Cyclin D1, and Cyclin E1 (58–60) showing that inhibiting O-GlcNAcylation negatively regulates NF-κB transcriptional activity (**Figure 7B**). Since the regulation of c-Myc, Cyclin D1, and Cyclin E1 largely account for the role of NF-κB in promoting cell growth (61), we studied the effect of O-GlcNAcylation inhibition on cell cycle progression in OCI-AML3 cells. We found that OSMI-1 treatment disrupted cell cycle progression and caused increased G1 arrest (**Figures 7C, D**) and decreased overall cell numbers (**Figure 7E**). These results indicate that increased O-GlcNAcylation promotes NF- κB activation and cell cycle progression in AML.

## 4 Discussion

While the dysregulation of major metabolic pathways such as glycolysis and oxidative phosphorylation in AML have been well defined (62), the role of HBP and O-GlcNAcylation as well as the expression of specific genes regulating this pathway in AML are less understood. Cancers display altered metabolism and enhanced glucose flux associated with HBP activity and O-GlcNAcylation (63). Increased O-GlcNAcylation has been linked to altered protein function that promotes the growth and survival of multiple cancer types including AML (45). Here, we studied both the single-cell and bulk RNA expression of key genes regulating the cycling of O-GlcNAcylation in primary patient samples in LSCs, blasts, and the bulk AML population. In addition, we assessed global O-GlcNAcylation and OGT protein expression in bulk and LSC AML populations by flow cytometry. Our findings show the HBP is dysregulated in AML and both AML blasts and LSCs show enhanced O-GlcNAcylation and OGT expression as compared to healthy controls. We also found both O-GlcNAcylation and OGT expression were relatively higher in AML blasts compared to LSCs, which suggest increased HBP activity may accompany the transition from LSCs to AML blasts. We also show AML cells exhibit enhanced expression of the enzymes controlling *de novo* UDP-GlcNAc synthesis. While previous studies have shown AML cell lines and bulk AML patient samples display increased O-GlcNAcylation levels (22), this study shows at the single-cell level O-GlcNAcylation as well as *OGT* and *OGA* expression are highly heterogeneous. The elucidation of this heterogeneity is important as strategies to target OGT and/or OGA will likely be impacted by its differential expression within patients. In addition, the expression of OGT/OGA and O-GlcNAcylation have not previously been reported in LSCs. LSCs are the critical component of AML responsible for AML initiation and relapse, and understanding of the expression patterns in these cells is important. As O-GlcNAcylation is upregulated in LSCs as compared to healthy controls, targeting O-GlcNAcylation may impair the survival/proliferation of LSCs.

Of note, it was somewhat surprising to see elevated HBP enzyme levels and O-GlcNAcylation in LSCs as they have previously demonstrated lower glycolysis rates and glucose uptake than healthy HSPCs (64). Because this would decrease HBP substrate availability, we would have expected to see that reflected with lower O-GlcNAcylation. Since we see LSCs have higher HBP activity and O-GlcNAcylation than HSPCs, this could potentially suggest LSCs maintain low glycolysis rates by diverting more glucose to the HBP. As low glycolysis rates are connected to maintaining quiescence, which limits their elimination by chemotherapeutics (64, 65), targeting LSC O-GlcNAcylation may represent a strategy to combat chemoresistance.

Our study also suggests increased O-GlcNAcylation in AML blasts and LSCs is in part due to the upregulation of enzymes regulating HBP in addition to the increased consumption of glucose and glutamine by cancer cells. In addition to the elevation in *de novo* HBP enzymes, both AML blasts and LSCs showed significantly upregulated *OGT*, but not *OGA*, suggesting amplified OGT function and minimal OGA function might be contributing to increased protein O-GlcNAcylation in AML.

Our analysis of *OGT* and *OGA* expression showed no difference between diagnostic and relapse samples suggesting mechanisms of relapse do not influence HBP enzyme expression patterns. Interestingly at the single-cell level, several clusters of cells expressed high levels of both *OGT* and *OGA*, and there was overall a strong correlation of *OGA* and *OGT* expression, which was also evident in cluster 3, where both enzymes were expressed at substantially lower levels than other clusters.

Our analysis shows *GFPT1* (GFAT1) is elevated in AML while *GFPT2* (GFAT2) is downregulated as compared to normal blood cells (**Figure 3B**). This suggests AML utilizes GFAT1 as the rate limiting enzyme for the HBP, unlike other cancers such as ovarian cancer (40, 65) and lung cancer (66), where GFAT2 appears to predominately drive the HBP. It is also interesting to note *NAGK* is downregulated in AML, suggesting a lower dependence of AML on the salvage arm of the HBP. This appears to be cancer specific as previously it has been shown NAGK expression is enhanced in pancreatic ductal adenocarcinoma and blocking NAGK leads to cancer cell death (39). It should be noted that due to limited availability of RNA-sequencing data from normal HSPCs, we were unable to perform properly powered statistical comparisons between AML cells and normal HSPCs. As more of this data becomes available, expression levels of HBP enzymes should be evaluated, as HSPCs will be a better healthy control than normal PBMCs. Another limitation with this study is that the majority of analysis, with the exception of focused flow cytometric analysis, is based on the analysis of RNA expression. While in many cases RNA and protein expression levels are correlated, it is possible that protein analysis may lead to different results in some cases.

Our data show genes involved in the UPR exhibit co-expression with *OGT* and *OGA*. In general, the UPR is induced by accumulation of improperly folded proteins in the ER and a major factor that prevents proper folding is insufficient N-linked glycosylation (67). Since UDP-GlcNAc is a major component of N-linked glycans, enhanced HBP in AML is expected to provide sufficient UDP-GlcNAc for N-linked glycosylation, thereby minimizing UPR activation. Therefore, enhanced co-expression of HBP and UPR genes appears to be regulated independently. As an alternate possibility, the UPR linked transcription factor Xbp1 was shown to upregulate HBP enzymes such as GFAT1, GNPNAT1, and PGM3 (68). This might contribute to UPR-induced enhanced O-GlcNAcylation, which in turn will allow survival of AML cells as increased O-GlcNAcylation abrogates the UPR induced apoptosis through disrupting the PERK-CHOP pathway (53). In contrast to this, it has also been shown that the UPR inhibits glucose metabolism and O-GlcNAcylation in neurons (69), which indicates a cell type specific role of the HBP. The possibility also exists where enhanced O-GlcNAcylation may enhance the UPR pathway, which remains to be explored.

The UPR has also shown to play an important role in promoting AML survival under conditions of both extrinsic and intrinsic stress. AML cells are subject to conditions of hypoxia and nutrient deprivation in the bone marrow microenvironment, both of which can disrupt proteostasis (70). In addition, AML cells produce high levels of reactive oxygen species (ROS), which causes proteotoxic stress (71). Because of this, UPR-targeted treatment modalities are being investigated as a therapeutic strategy for AML (72). Thus, the relationship between O-GlcNAcylation and the UPR in AML is an area that warrants further investigation to better understand the response to these treatments.

This study shows that one way which hyper-O-GlcNAcylation promotes AML cell growth is through positively regulating NF-κB activity. It has been previously shown that O-GlcNAcylation regulates p65 nuclear translocation by inhibiting its interaction with IκBα (46). Our study shows that, in addition to p65, enhanced O-GlcNAcylation also promotes c-Rel and p50 nuclear translocation (**Figure 7A**). Although p50 O-GlcNAcylation has not been well-characterized, it is possible that both direct O-GlcNAcylation of p50 as well as its dimerization with O-GlcNAcylated p65 or c-Rel may regulate its nuclear translocation. It remains to be determined whether the homodimer of p50/p50 that primarily acts as a transcriptional repressor (73) is O-GlcNAcylated in AML, which may alter its repressive function. Overall, it appears that elevated HBP activity and O-GlcNAcylation likely supports constitutive NF-κB signaling in AML patients alluding to the therapeutic potential of targeting NF-κB O-GlcNAcylation as a promising approach to treat AML. Current treatments targeting NF-κB in AML include proteasome inhibitors such as bortezomib, which shows pan-NF-κB inhibitory function (74). Bortezomib has shown promising potential to eliminate AML cells, particularly when used in combination with chemotherapeutic agents (75, 76). However, adverse side effects have been observed probably because the broad inhibition of NF-κB compromises its diverse roles in healthy cells (77). Hence, specifically targeting NF-κB O-GlcNAcylation may offer an alternative approach to inhibit aberrant NF-κB activity in AML and other cancers while preserving its O-GlcNAcylation-independent biological functions of that pathway. Moreover, as constitutive NF-κB activation has been shown to occur at higher levels in LSCs than in HSPCs (78), blocking NF-κB O-GlcNAcylation holds potential to selectively eliminate LSCs, to prevent AML progression and relapse.

Progress in the improvement in outcomes for AML has been modest for the past 40 years for most AML patients (4, 79). Factors such as specific cytogenetic abnormalities or mutations greatly influence both the choice and outcome of current treatments. It is unclear how different combinations of mutations and cytogenetic abnormalities may contribute to the overall role of O-GlcNAcylation in AML. Hence, future studies should focus on characterizing how AML heterogeneity influences the effects of O-GlcNAcylation on specific subsets of AML survival and proliferation. Discovery of novel pathways and molecular mechanisms, which may function across AML subtypes are important to fill the gap in knowledge to develop treatment strategies to control AML progression. This study provides a significant advancement on the role of the HBP in AML at global and single cell levels and reveals it is enriched across AML subtypes and AML differentiation status. However, this study was limited in exploring the mechanistic effect of O-GlcNAcylation across multiple AML subtypes and offers only a biased functional perspective with OCI-AML3 cells, which represents a distinct AML subtype. Future studies should focus on characterizing how AML heterogeneity influences the effects of O-GlcNAcylation on AML survival and proliferation. In addition, although this study identified multiple signaling pathways such as TGF-β, MAPK, Wnt, and JAK/STAT that are enriched with enhanced expression of O-GlcNAc cycling enzymes, these pathways were not explored from a functional perspective. Although, O-GlcNAcylation has previously been shown to regulate these pathways in general, further studies to understand their specific O-GlcNAcylation-dependent regulation in the context of AML is needed.

The dynamics of O-GlcNAcylation are linked to glucose metabolism. It appears to act as a connecting link between the metabolic status of the cell, especially the glycemic status, and aberrant protein and cellular functions. Moreover, chemotherapeutic drugs such as doxorubicin and camptothecin have been shown to enhance O-GlcNAcylation and activate cell survival pathways (23). Combination of HBP inhibitors with chemotherapeutic drugs has been shown to yield better outcome in limiting cancer cell growth (23).

Similar to glucose, availability of glutamine also feeds into the HBP—enhancing cellular O-GlcNAcylation (80). LSCs are more reliant on amino acid, i.e., glutamine, metabolism than HSPCs to fuel oxidative phosphorylation for their survival (53). Dependence of LSCs on amino acid metabolism was also shown at the single-cell level (11), suggesting both glucose and glutamine metabolism may regulate the HBP and O-GlcNAcylation in AML LSCs. Hence, targeting metabolism-dependent protein modification such as O-GlcNAcylation offers a promising approach, that may be used as a monotherapy or in combination with other treatments, toward developing therapeutics for AML as well as several other cancers where deregulated glucose metabolism and hyper O-GlcNAcylation exists as a hallmark.

## Data availability statement

Publicly available datasets were analyzed in this study. All the data are included in the main figures and Supplementary Materials. Code is available at GitHub at the repository: rschauner/2024-frontiers-immunology-aml-hbp. Scripts to download the data are found as part of the GitHub repository and in the README. Datasets include: GTEx (via www.gtexportal.org); TCGA, BeatAML, and TARGET (via GDC commons); St. Jude Cloud (via platform.stjude.cloud/data, accession numbers SJC-DS-1013 and SJC-DS-1009) and GSE198919, GSE116256, and GSE126068 via GEO). Further inquiries can be directed to the corresponding authors. 

## Ethics statement

This study involved human cells obtained from the Case Comprehensive Cancer Center Hematopoietic Biorepository and Cellular Therapy core facility and was approved by the University Hospitals Cleveland Medical Center IRB. The studies were conducted in accordance with the local legislation and institutional requirements. No participants were recruited for this study.

## Author contributions

RS: Data curation, Formal Analysis, Investigation, Methodology, Visualization, Writing – original draft, Writing – review & editing, Software. JC: Formal Analysis, Methodology, Validation, Writing – original draft, Writing – review & editing, Investigation, Visualization. CH: Data curation, Formal Analysis, Writing – review & editing. DW: Formal Analysis, Methodology, Validation, Writing – original draft, Writing – review & editing, Conceptualization, Data curation, Funding acquisition, Project administration, Resources, Supervision. PR: Data curation, Formal Analysis, Conceptualization, Funding acquisition, Investigation, Methodology, Project administration, Resources, Supervision, Validation, Visualization, Writing – original draft, Writing – review & editing.

## Supplementary material

Supplementary Figure 1 Flow cytometry gating strategy. **(A)** Gating strategy for AML samples including LSCs and Bulk AML cells. **(B)** Gating strategy for healthy donor HSPCs.

Supplementary Figure 2 Flow cytometric validation. **(A)** Histograms for O-GlcNAc levels for each patient and cell-type. **(B)** Western blot analysis of OCI-AML3 cells with Thiamet G or OSMI-1. β-Tubulin used as a loading control. **(C)** Flow cytometry dot plot showing co-staining of O-GlcNAc and OGT.

Supplementary Table 1 Per cluster DE for HBP genes.

Supplementary Table 2 Sheet 1: DE between AML samples and Whole Blood in bulk RNA-sequencing. Sheet 2: DE between adult and pediatric in bulk RNA-sequencing data.

Supplementary Table 3 GSVA Results for OGT high v. low.

Supplementary Table 4 GSVA Results for OGA high v. low.

Supplementary Table 5 Pearson correlation coefficients for OGT and RL2 MFI.
