# Supplementary figures and images for: Single cell and bulk RNA expression analyses identify enhanced hexosamine biosynthetic pathway and O-GlcNAcylation in acute myeloid leukemia blasts and stem cells
Source: Front Immunol. 2024 Mar 27;15:1327405. doi: 10.3389/fimmu.2024.1327405 (PMC11004450; doi:10.3389/fimmu.2024.1327405)

**A**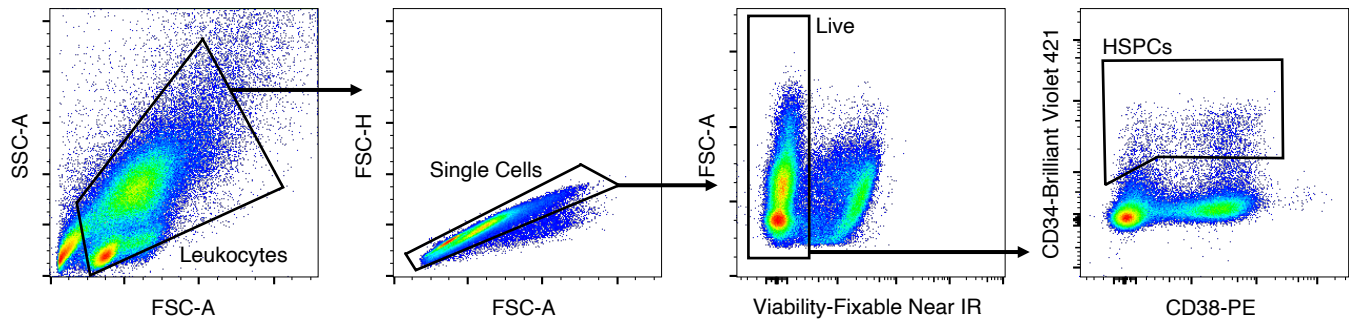**B**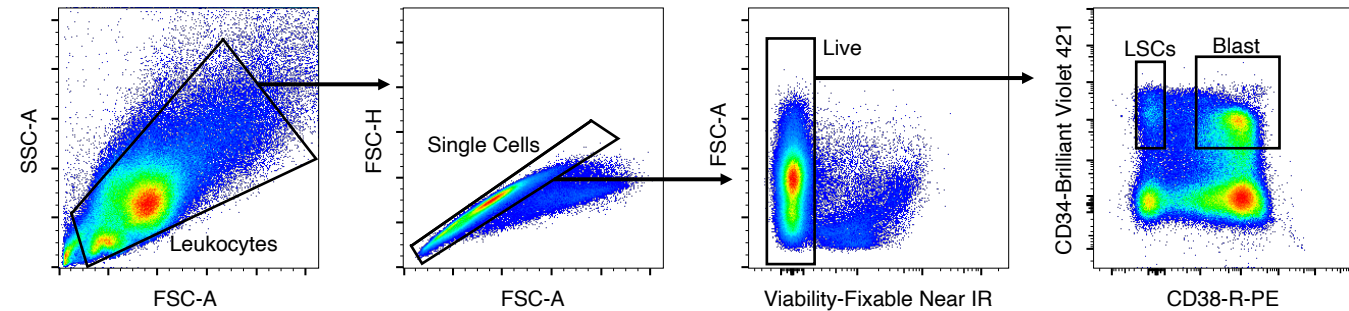

Supplement: Supplementary Figure 1 — Flow cytometry gating strategy. (A) Gating strategy for AML samples including LSCs and Bulk AML cells. (B) Gating strategy for healthy donor HSPCs. [file DataSheet_1.pdf]

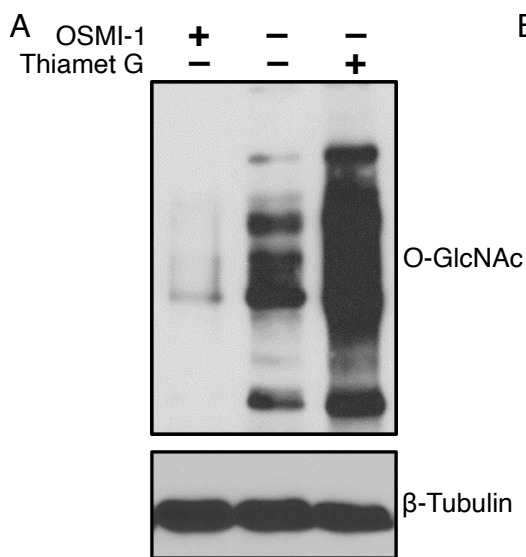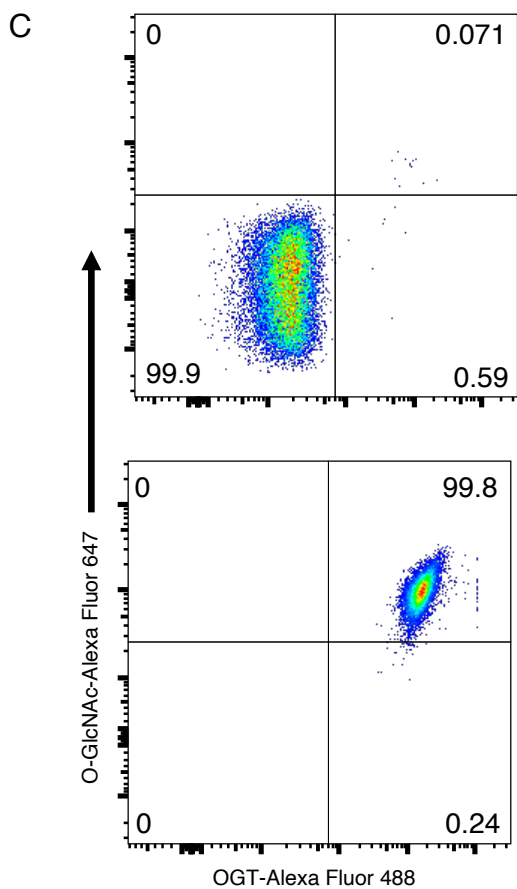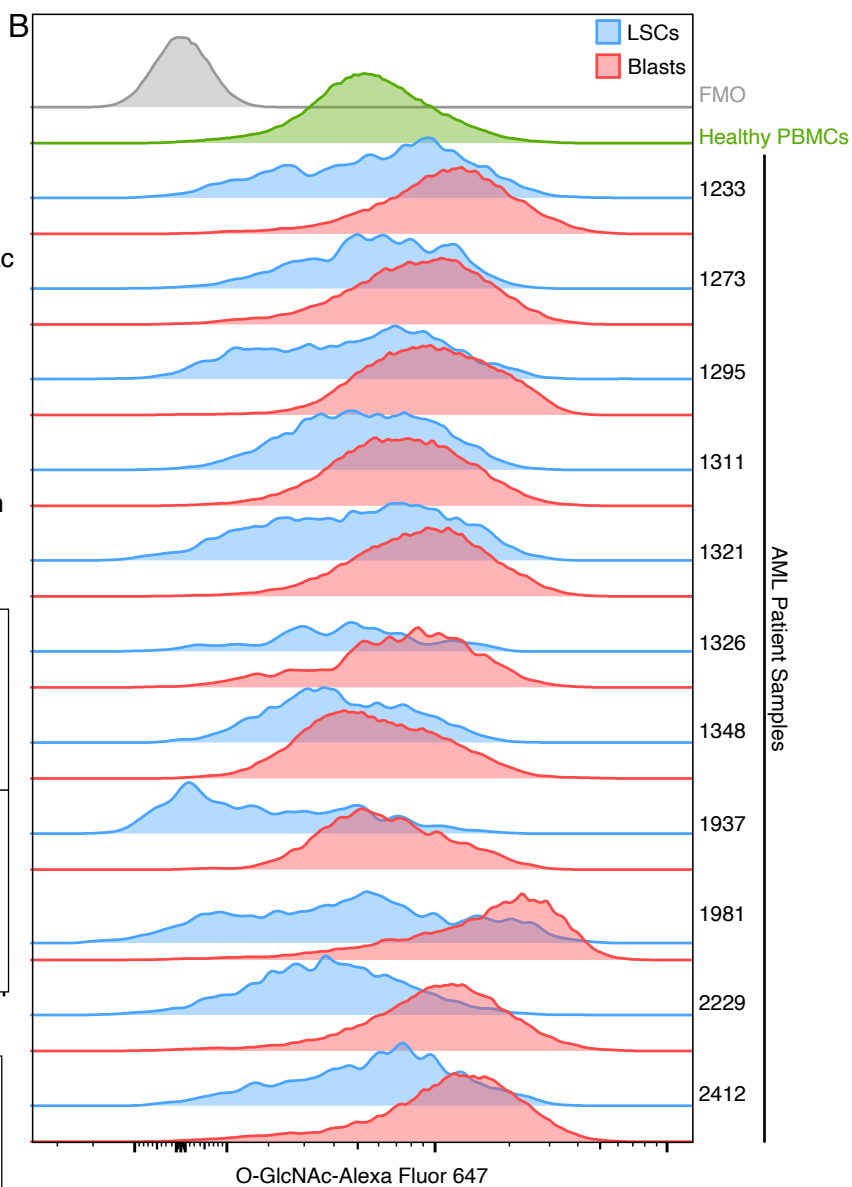

Supplement: Supplementary Figure 2 — Flow cytometric validation. (A) Histograms for O-GlcNAc levels for each patient and cell-type. (B) Western blot analysis of OCI-AML3 cells with Thiamet G or OSMI-1. β-Tubulin used as a loading control. (C) Flow cytometry dot plot showing co-staining of O-GlcNAc and OGT. [file DataSheet_2.pdf]
